# A rare coincidence of different types of driver mutations among uterine leiomyomas (UL)

**DOI:** 10.1186/s13039-015-0177-9

**Published:** 2015-10-14

**Authors:** Carsten Holzmann, Dominique Nadine Markowski, Sabine Bartnitzke, Dirk Koczan, Burkhard Maria Helmke, Jörn Bullerdiek

**Affiliations:** Institute of Medical Genetics, University Rostock Medical Center, Ernst-Heydemann-Strasse 8, D-18057 Rostock, Germany; Center of Human Genetics, University of Bremen, Leobener Strasse ZHG, D-28359 Bremen, Germany; Institute of Immunology, University of Rostock, University Rostock Medical Center, Schillingallee 70, D-18057 Rostock, Germany; Institute of Pathology, University of Heidelberg, Heidelberg, Germany; Present address: Institute of Pathology, Elbe Kliniken, Klinikum Stade, Bremervörder Str. 111, D- 21682 Stade, Germany

**Keywords:** Uterine leiomyoma, Genetic types, HMGA2, MED12, MED12L

## Abstract

Mutations of *mediator subcomplex 12* (*MED12*) and of high mobility group protein AT-hook 2 (*HMGA2*) are driver mutations in uterine leiomyomas (UL) that have not been observed to coexist in one tumor and even rarely coexist in different UL tumors of one patient. Here we describe a patient who underwent hysterectomy because of multiple leiomyomas which were studied by cytogenetics, *MED12* hotspot sequencing, and copy number variation arrays. Two of the UL tumors had different *HMGA2* rearrangements not detected by G-banding. Two UL tumors had deletions of the long arm of chromosome 3, in one case associated with a *MED12* mutation. Both deletions lead to the loss of *MED12L* showing strong similarity with *MED12*. It remains to be determined if this gene can play a role in leiomyomagenesis independent of *MED12*. In summary, the patient presented exhibits an unusual coincidence of different driver mutations among her leiomyomas.

**Electronic supplementary material:**

The online version of this article (doi:10.1186/s13039-015-0177-9) contains supplementary material, which is available to authorized users.

## Background

Uterine leiomyomas (UL) are likely to constitute the most frequent symptomatic human tumors at all. Despite a significant morbidity they can cause, the associated health disparities as well as the enormous costs related to the disease surprisingly little is known about the roots of leiomyoma development. Statistical correlations and theories have been advanced to explain these frequent and clinically highly relevant neoplasms. Somatic alterations of the tumor genome still can be expected to give important novel insights thus representing a solid base for a better understanding.

As to their molecular pathogenesis UL present as a heterogeneous group of diseases due to different driver mutations part of which can be detected by cytogenetic investigations while other somatic mutations are restricted to single base exchanges, small deletions and small insertions [1, 2]. Of these, those affecting the genes encoding mediator subcomplex 12 (*MED12*) and high mobility group protein AT-hook 2 (*HMGA2*) apparently characterize two independent types of UL [3–5]. In contrast to other genetic abnormalities non-randomly seen in UL, both these genetic alterations have not been observed to coexist within one tumor and even their coexistence in different UL tumors of one patient appears to be a very rare finding [6]. Recently, evidence has been presented that UL tumors with these mutations also differ in their clinical behavior with e.g. *HMGA2*-rearranged UL presenting with a larger average size than those with *MED12* mutations [4, 6]. Also, they tend to occur as solitary nodules [6] whereas often multiple clonally independent leiomyomas with *MED12* mutations have been described. Furthermore, the literature holds several examples of leiomyosarcomas and STUMP (smooth muscle tumors of uncertain malignant potential) carrying *MED12* mutations indistinguishable from those found in “ordinary” UL [7–13]. These latter cases suggest a rare but existing leiomyoma - STUMP - leiomyosarcoma sequence likely depending on the occurrence of further genetic alterations in addition to the driver mutation of *MED12*. In contrast, neither STUMP nor uterine leiomyosarcomas with *HMGA2* alterations akin to those seen in UL have been reported so far. However, considering the ongoing discussion of the risk of tumor spread due to power morcellation, any attempts to gain further insights into the molecular pathogenesis of malignant transformation within UL are of high interest.

As to alterations of the two genes *MED12* and *HMGA2* there is ample evidence that we really deal with two pathogenetically and clinically distinct tumor entities. Here we describe the results of our genetic studies on UL of a woman apparently carrying both types of tumors along with those having other driver mutations. The implications of our findings with respect to the pathogenetic relevance of different driver mutations will be discussed.

## Case presentation

A 48 year old patient underwent laparotomic hysterectomy because of symptomatic uterine leiomyomas. After hysterectomy, gross examination revealed the presence of four leiomyomas ranging in diameter between 3 cm and 8 cm. Histologic examination of all four UL tumors revealed typical benign smooth muscle tumors NOS (“not otherwise specified”) without evidence for UL variants or STUMP lesions. Shortly after hysterectomy the patient was diagnosed with an ER/PR-negative breast cancer that showed overexpression of HER2/neu, but one year after hysterectomy no evidence for recurrence of uterine tumors, as e.g. peritoneal spreading, was obtained.

**Fig. 1:**
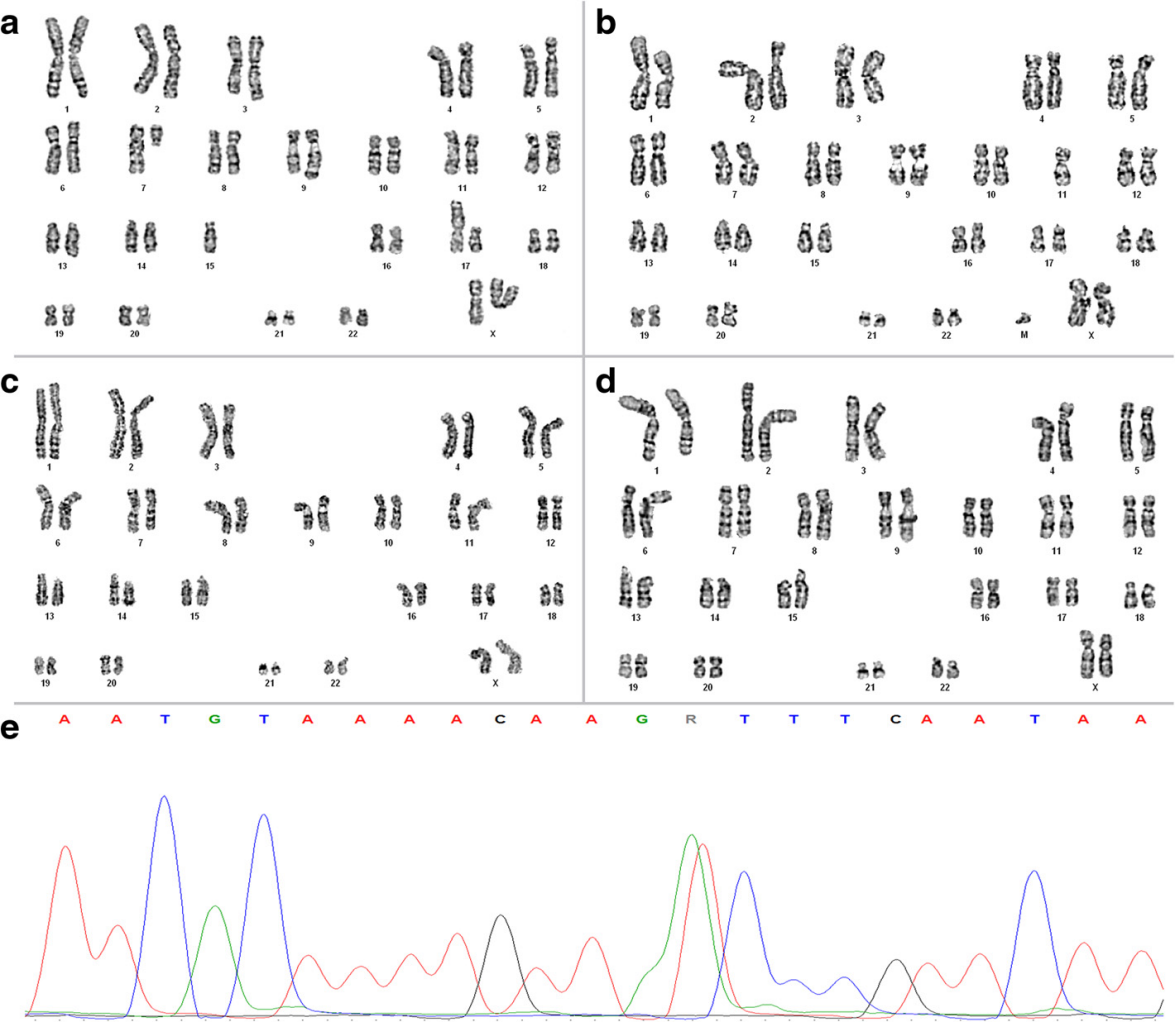
Cytogenetic and molecular analysis of uterine leiomyomas. **a**-**d** representative G-banded karyotypes of four uterine leiomyomas. **a** tumor 709/1, Karyotype: 45,XX,del(3)(q24q25.33),del(7)(q11.2),add(9)(q34),-15,der(17)t(15;17)(q?22;p?11.2)[3]/46,XX[3]. Of ten additional hypodiploid metaphases seven showed structural abnormalities and losses and three only losses of chromosomes. In contrast to the structural abnormalities, none of the losses observed were clonal. **b** tumor 709/2, karyotype: 43 ~ 46,XX,del(1)(p34),der(2),-11 + mar. **c** tumor 709/3, karyotype: 46,XX[22]. **d** tumor 709/4, karyotype: 46,XX[17]. **e** mutation of *MED12* (c.131G > A) of tumor 709/2

**Fig. 2:**
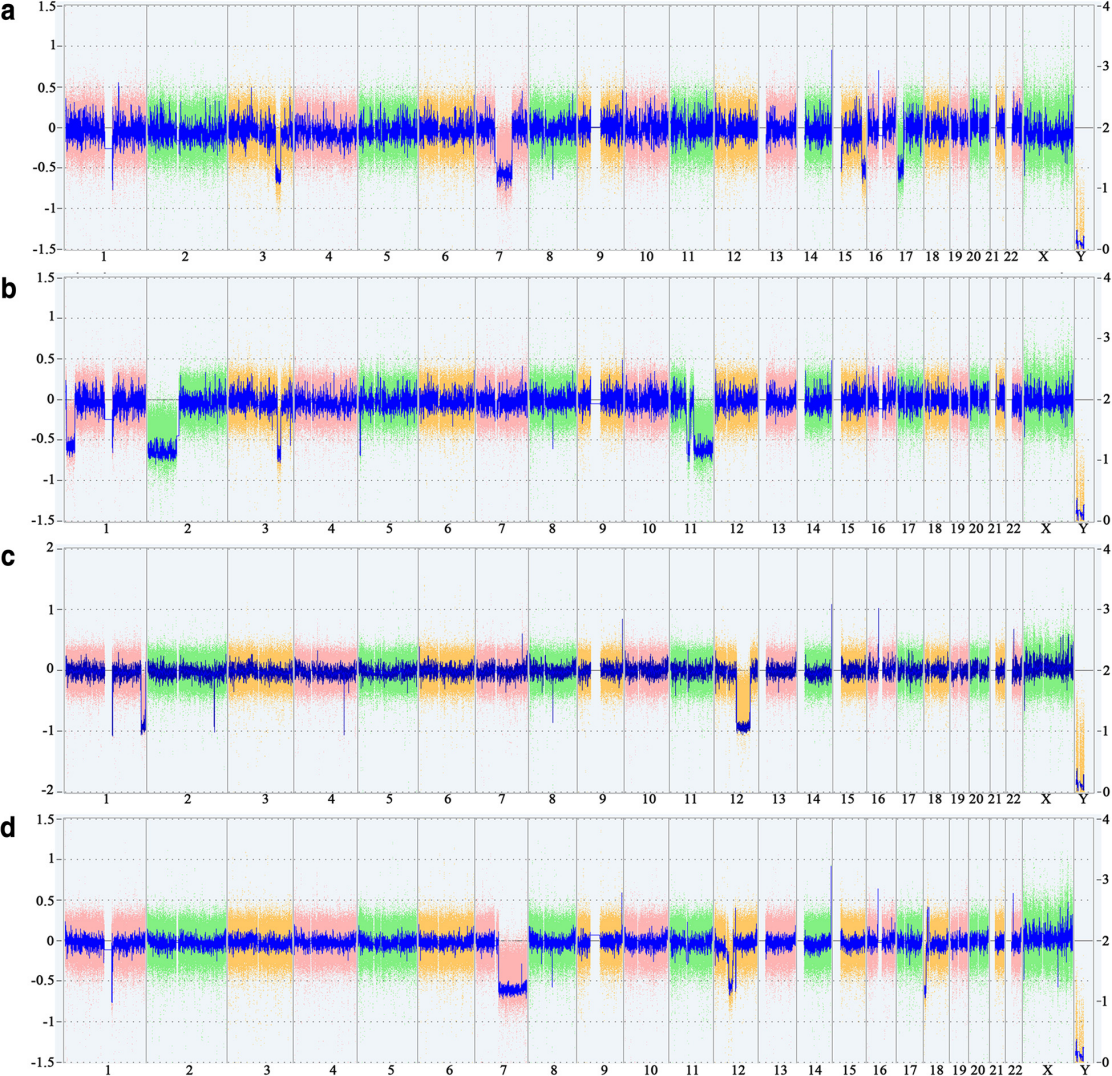
Results of CNV-array-analyses. Whole genome views of four uterine leiomyomas investigated. The weihghted log2 ratio of the probes are displayed as colored dots, the smooth signal (Gaussian smoothed calibrated copy number estimate) is displayed as blue line. **a** tumor 709/1. **b** tumor 709/2. **c** tumor 709/3. **d** tumor 709/4

**Fig. 3:**
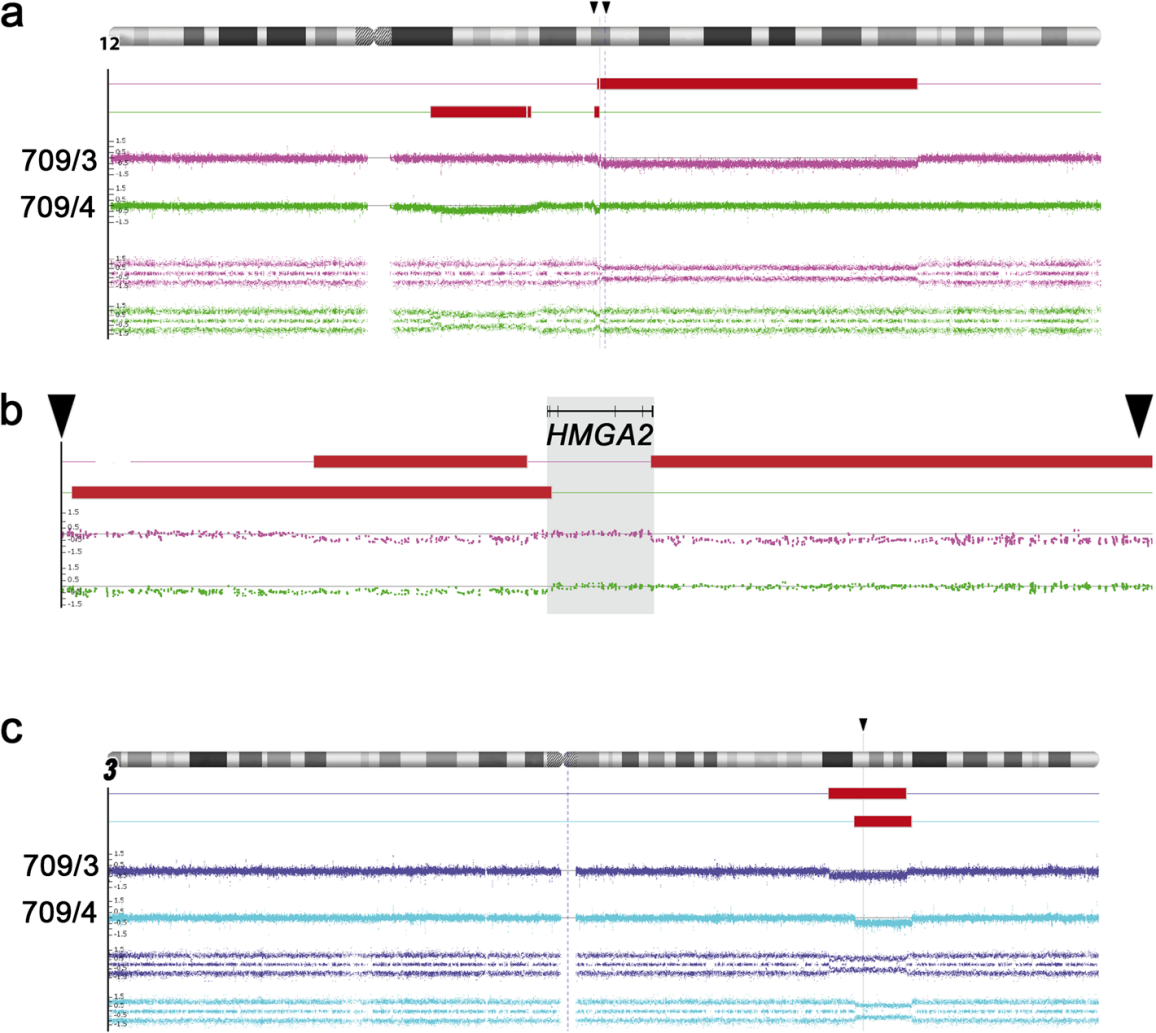
Rearrangements of chromosome 12 and deletions of the long arm of chromosome 3. **a** losses of parts of chromosome 12 in tumors 709/3 and 709/4. Black arrowheads indicate the region enlarged in (**b**). **b**. position of the *HMGA2* gene with intron/exon structure as indicated in a. **c**. Position of interstitial deletions of the long arm of chromosome 3 in tumors 709/1 and 709/2, respectively. The grey line and a black arrowhead indicate the position of the *MED12L* gene

Pieces of four UL tumors were obtained from the hysterectomy specimen. Molecular and cytogenetic analyses of these four UL tumors were carried out. Cytogenetic analyses failed to detect clonal karyotypic aberrations in two of the UL tumors (UL 709/3 and 709/4. Fig. 1a-d, Additional file 1: Table S1) and one of the UL tumors carried a *MED12* mutation (709/2. c.131G > A, Fig. 1e). In contrast to the results obtained by G-banding, the CNV arrays revealed more or less gross genomic alteration in all four UL tumors (Fig. 2) that in neither of the UL tumors could have escaped detection by G-banding. E.g., in two UL tumors losses within the close surrounding of or within the *HMGA2* locus point to rearrangements of this gene (Fig. 3a, b). As other genomic alterations frequently seen in UL, two of the UL tumors were characterized by deletions of the long arm of chromosome 7 with different sizes.

**Fig. 4:**
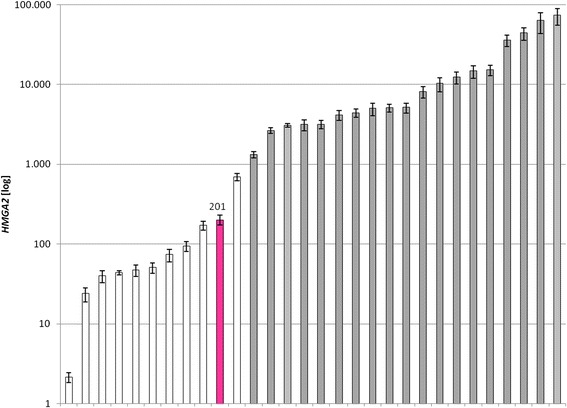
Expression of HMGA2 mRNA in fibroid 709/3 as compared to ten other arbitrarily chosen fibroids without cytogenetically detectable rearrangements of chromosomal region 12q14-15 (white columns) as well as to 19 fibroids that showed a microscopically visible rearrangement of this chromosomal segment (grey columns), respectively

Of note, UL 709/3, presented with two deletions located on either side of the *HMGA2* locus. Of these, a short 5′proximal deletion mapped outside the putative transcription start of the gene removing a segment of only approximately 280 kbp (Fig. 3a). A much larger deletion of roughly 42.79 Mb had removed part of the 3′UTR of the gene. In terms of G-banding this latter deletion resulted in the partial monosomy for the bands 12q14.3-q24.11. *HMGA2* mRNA expression analysis carried out in this case revealed a slightly elevated expression compared to other UL without clonal cytogenetic abnormalities but was clearly lower than in cases with cytogenetically apparent rearrangement of the 12q14-15 region (Fig. 4). As not uncommon in UL with *HMGA2* rearrangements this tumor in addition had a deletion of part of a terminal region of the long arm of chromosome 1 with a size of approximately 14.11 Mb affecting chromosomal bands 1q42.2-q44 including the *fumarate hydratase* gene (*FH*). Both the large deletion of 12q as well as those of 1q should be detectable by G-banding but neither clonal karyotypic abnormalities were seen in this case nor was even a single metaphase seen presenting with these abnormalities. The second UL (709/4) with a putative *HMGA2* rearrangement presented with deletions that both mapped proximal to the gene locus again including one large and one smaller deletion. The large deletion had a size of approximately 13.44 Mb and mapped within chromosomal region 12q12-q13.3 whereas the small deletions had a size of only approximately 630 kbp and mapped in the direct vicinity of *HMGA2*. From the array results it cannot even be ruled out that by this deletion part of the exons 1 and 2 of the gene had been removed (Fig. 3b). In addition, this UL carried three deletions on the long arm of chromosome 7 that were interspersed with short non-deleted fragments leading to a total loss of roughly 89,05 Mb from chromosomal region 7q11.22-q36.3. In this case as well, neither evidence for the presence of chromosomal alterations in general nor for those involving the deleted regions as detected by the array analysis was obtained. Thus, as the most likely explanation, in both UL tumors affected by *HMGA2* rearrangements the tumor cells did escape detection by G-banding due to their reduced ability or even inability to proliferate *in vitro*.

Of the two UL tumors presenting no evidence for a *HMGA2* rearrangements as detected by G-banding or array analysis (709/1, 709/2) one had a *MED12* mutation but both showed large deletions of the short arm of chromosome 3. Of note, the deleted segments in both UL tumors caused a removal of the gene encoding *MED12L* (Fig. 3c). In addition, both UL carried other large deletions that, in these both UL tumors akin to the deletions of 3p, did not escape detection by G-banding (Tab.1). However, of note in case of 709/2 with the *MED12* mutation the cytogenetic preparation resulted in only two metaphases which could be investigated. Both shared cytogenetic abnormalities that thus can be considered being clonal (Fig. 1).

**Table 1:** Karyotypes and culture conditions of the tumors

Tumor no	Karyotype according to ISCN	Days in culture until cytogenetic preparation
709/1	45,XX,del(3)(q2?),del(7)(q11.2),add(9)(q34),-15,der(17)t(15;17)?(q22;p11.2)[3]/46,XX[3]	14
	Ten additional metaphases were hypodiploid. Of these, seven showed structural abnormalities and losses and three only losses of chromosomes. None of the losses observed were clonal.	
709/2	43 ~ 46,XX,del(1)(p34)[[2],der(2)[2],-11[2],+mar[2][cp2]	35
709/3	46,XX[22]	35
709/4	46,XX[17]/44,XX,-19,-21[1]	20

## Conclusions

Among the few putative driver mutations observed in UL those affecting *MED12* and these leading to rearrangements of *HMGA2* are particularly frequent and so far never have been reported to coincide within one individual tumor. Also, patients harboring UL of both types seem to be very rare. The present case deals with one such coincidence. Being studied by G-banding as well as by molecular analyses i.e. *MED12* sequencing and CNV arrays all four UL tumors revealed individual patterns of genomic alterations indicating their independent clonal origin. Simultaneously, a rare coincidence of several putative driver mutations displayed by the single tumor was noted.

In two of them the profiles point to rearrangements of *HMGA2* that had escaped detection by G-banding. In general, two explanations are possible for this lack of detection. First, the underlying rearrangements may have occurred at a submicroscopic level. This explanation likely does not fit because the large deletions accompanying both alterations are in a range easily detectable even when only applying a low resolution of chromosomal bands. Alternatively, the findings can be explained by a reduced or even absent ability of the affected cells to proliferate *in vitro*. Such a reduced ability recently has been observed for cell cultures of *MED12* mutated UL [14]. Nevertheless, in that study cells of *HMGA2*-mutated UL were able to proliferate for numerous *in vitro* passages. At a first glance, this finding seems to contradict the latter explanation for the absence of metaphases with the said deletions. In the present study, however, both UL tumors with presumed *HMGA2* rearrangements did also show other apparently independent abnormalities i.e. a large deletion of the long arm of chromosome 1 in UL 709/3 and a large deletion of the long arm of chromosome 7 in UL 709/4. Both abnormalities have been described in UL before either in the presence or in the absence of structural rearrangements of chromosome 12 [15–17]. Likewise, deletions of the long arm of chromosome 7 can by accompanied by *MED12* mutations or occur independently [3]. In our recent study on the *in vitro* proliferation of UL cells the vitro “long term survivors” did not show one of these abnormalities [14]. It seems reasonable to assume that, independent of their coincidence with other driver mutations, both abnormalities reduce *in vitro* proliferation. In turn, the actual frequency of *HMGA2* rearrangements may be underestimated when based solely on cytogenetics. In case of reduced ability to proliferate, whole genome sequencing [4] as well as genomic array analysis seem to be more efficient to detect these rearrangements if they are accompanied by simultaneous losses of chromosomal material. Of note, even when showing cytogenetic abnormalities of 12q14-15 microscopic analyses may not reflect the complexity of underlying *HMGA2* rearrangements sufficiently. Vice versa, the mere number of genetic imbalances as such does not allow diagnosing malignant growth [18].

Another interesting aspect in the UL tumors presented herein relates to the deletion of the long arm of chromosome 3 found in two UL tumors including one without detectable rearrangements of *HMGA2* or *MED12* mutations. Deletions of the long arm of chromosome 3 repeatedly have been described before in UL. In their study on 52 uterine leiomyomas with clonal chromosome abnormalities, Dal Cin P, Moerman P, Deprest J, Brosens I, Van den Berghe H. identified eight tumors with cytogenetic alterations that did not fit with any well-delineated cytogenetic subgroup [19]. In three of these cases the long arm of chromosome 3 was involved. Accordingly, it was considered that these changes characterize a new cytogenetic subgroup of uterine leiomyomas. Given that these deletions all point to the same target gene a loss-of-function for genes in that region as e.g. *HLTF*, *SIAH2*, *RAP2B*, *MME*, *GMPS*, *MLF1* und *RARRES1* should be considered. On the other hand in both UL tumors presented herein *MED12L* mapped within the region of overlap. The strong similarity between both genes and their proteins makes it reasonable to consider an independent role of alterations of that gene for the molecular pathogenesis in rare cases of UL.

In summary, the present case demonstrates the rare occurrence of a marked genetic heterogeneity among uterine leiomyomas with every single tumor displaying a unique CNV pattern. Loss of heterozygosity affecting the *MED12L* locus represents a candidate of a possible novel driver mutation in UL. Given the low but albeit existing probability of malignant transformation within UL the results point to the number of UL tumors as a risk factor associated with power morcellation of uterine tumors.

## Methods

### Tumor samples

From each of the UL tumors (lab code 709/1 - 709/4) one part was fixed for histologic examination, another part was snap frozen in liquid nitrogen, and a third part was kept in Hank’s solution for subsequent cell cultures.

### Histologic examination

The tumors were fixed in paraformaldehyde (4% in PBS) and processed for paraffin embedding. Tissue sections (1–2 μm thickness) were deparaffinized in xylene, rehydrated through a series of ethanol, and stained with hematoxylin and eosin (H&E).

### Cytogenetic studies

Chromosome analyses of cell cultures were performed following routine techniques as described earlier [20].

### DNA isolation

For CNV analysis as well as *MED12* mutation analysis DNA from the frozen tissue samples was isolated using the QIAamp DNA Mini Kit (Qiagen, Hilden, Germany) on a QIACube (Qiagen) according to the manufacturer’s instructions. The amount of double stranded DNA was measured using the Qubit dsDNA HS Assay Kit and a Qubit Fluorometer (Life Technologies, Carlsbad, CA).

### PCR and sequencing

For PCR amplification 100 ng of genomic template DNA were used. Primers to amplify the desired human PCR fragment of the *MED12* gene were those recently described [15]. Subsequently, PCR-products were separated by agarose gel-electrophoresis and the desired DNA-fragments/-bands were extracted by a QIAquick Gel Extraction Kit (Qiagen) using a QIACube (Qiagen) according to manufacturer’s instructions. DNA-sequencing of the purified PCR-products was performed using the CEQ DTCS Quick Start Sequencing Kit and a Beckman Coulter GenomeLab GeXP Genetic Analysis System (AB SCIEX, Framingham, MA, USA).

### Arrays

CNV (copy number variation) analysis was performed using premade CytoScan HD Arrays (Affymetrix, Santa Clara, CA) consisting of more than 2.4 million markers for copy number and approximately 750,000 single nucleotide polymorphisms (SNPs). Enriched gene coverage for cancer and constitutional genes results in marker- base ratio coverages of 1/384 for ISCA, 1/553 for cancer genes, 1/486 for X-chromosomal genes and 1/659 for 12,000 OMIM genes. Labelling of 250 ng DNA and hybridization were done following the manufacturer’s instructions. After staining and washing using a Gene-Chip Fluidics Station 450 (Affymetrix) the arrays were scanned by an Affymetrix 3000 7G scanner. Arrays were analyzed through the Affymetrix Chromosome Analysis Suite (ChAS) software (ChAS analysis files for CytoScan® HD Array version NA33). Numbering of map positions was based on hg19 (NCBI Build 37 reference sequence).

### Quantification of HMGA2 mRNA

Expression of *HMGA2* mRNA was analysed as described earlier [21]. Briefly, RNA isolated from the samples had been was digested by DNAse and then used for cDNA synthesis. For quantification by real-time PCR (Applied Biosystems 7300, Applied Biosystems, Darmstadt, Germany), a commercially available gene expression assay (Hs00171569, Applied Biosystems) was used. *HMGA2* mRNA expression was quantified relatively to *HPRT* mRNA, which was used as an endogenous control.

## Consent

The study was approved by the local ethics committee. Samples were obtained in accordance with the declaration of Helsinki and informed written consent was obtained from the patient prior to surgery.

## Additional file

Additional file 1: Table S1. Results of array analysis. Overview of copy number variations with 200 kb minimal size. (DOCX 29 kb)
