# Supplementary material for: A rare coincidence of different types of driver mutations among uterine leiomyomas (UL)
Source: Mol Cytogenet. 2015 Oct 14;8:76. doi: 10.1186/s13039-015-0177-9 (PMC4604635; doi:10.1186/s13039-015-0177-9)
Supplement: Additional file 1: Table S1. — Results of array analysis. Overview of copy number variations with 200 kb minimal size. (DOCX 29 kb) [file 13039_2015_177_MOESM1_ESM.docx]

| Tumor | CN State | Type | Chromosome | Cytoband Start | Cytoband End | Size (kbp) | Gene Count |
| --- | --- | --- | --- | --- | --- | --- | --- |
| MY709.1 | 1.0 | Loss | 1 | q21.1 | q21.1 | 520.498 | 21 |
| MY709.1 | 1.0 | Loss | 1 | q21.1 | q21.1 | 520.498 | 21 |
| MY709.1 | 1.0 | Loss | 1 | q21.1 | q21.1 | 391.213 | 5 |
| MY709.1 | 3.0 | Gain | 1 | q23.3 | q23.3 | 204.298 | 2 |
| MY709.1 | 1.0 | Loss | 3 | q24 | q24 | 3027.87 | 5 |
| MY709.1 | 1.0 | Loss | 3 | q24 | q25.33 | 15.523.205 | 88 |
| MY709.1 | 1.0 | Loss | 3 | q24 | q25.33 | 12.456.448 | 82 |
| MY709.1 | 1.0 | Loss | 7 | q11.21 | q11.23 | 10.997.197 | 79 |
| MY709.1 | 1.0 | Loss | 7 | q11.21 | q11.23 | 10.997.197 | 79 |
| MY709.1 | 1.0 | Loss | 7 | q11.23 | q31.1 | 37.041.248 | 324 |
| MY709.1 | 1.0 | Loss | 7 | q11.23 | q31.1 | 37.041.248 | 324 |
| MY709.1 | 1.0 | Loss | 11 | q11 | q11 | 307.844 | 0 |
| MY709.1 | 3.0 | Gain | 14 | q32.33 | q32.33 | 513.628 | 3 |
| MY709.1 | 3.0 | Gain | 14 | q32.33 | q32.33 | 513.628 | 3 |
| MY709.1 | 1.0 | Loss | 15 | q11.2 | q11.2 | 596.755 | 7 |
| MY709.1 | 1.0 | Loss | 15 | q11.2 | q11.2 | 269.361 | 2 |
| MY709.1 | 1.0 | Loss | 15 | q25.3 | q26.1 | 3.577.246 | 53 |
| MY709.1 | 1.0 | Loss | 15 | q25.3 | q26.3 | 13.279.752 | 99 |
| MY709.1 | 1.0 | Loss | 15 | q26.1 | q26.1 | 917.617 | 9 |
| MY709.1 | 1.0 | Loss | 15 | q26.1 | q26.3 | 5.623.222 | 14 |
| MY709.1 | 1.0 | Loss | 15 | q26.3 | q26.3 | 537.756 | 6 |
| MY709.1 | 1.0 | Loss | 15 | q26.3 | q26.3 | 2.243.887 | 18 |
| MY709.1 | 3.0 | Gain | 16 | p11.2 | p11.1 | 284.519 | 3 |
| MY709.1 | 3.0 | Gain | 16 | p11.2 | p11.1 | 284.519 | 3 |
| MY709.1 | 1.0 | Loss | 17 | p13.3 | p13.3 | 801.144 | 11 |
| MY709.1 | 1.0 | Loss | 17 | p13.3 | p11.2 | 17.474.555 | 334 |
| MY709.1 | 1.0 | Loss | 17 | p13.3 | p13.3 | 940.527 | 22 |
| MY709.1 | 1.0 | Loss | 17 | p13.3 | p13.2 | 1.530.588 | 31 |
| MY709.1 | 1.0 | Loss | 17 | p13.2 | p13.1 | 3526.56 | 89 |
| MY709.1 | 1.0 | Loss | 17 | p13.1 | p12 | 4.856.624 | 92 |
| MY709.1 | 1.0 | Loss | 17 | p12 | p12 | 538.504 | 3 |
| MY709.1 | 1.0 | Loss | 17 | p12 | p12 | 2.239.269 | 11 |
| MY709.1 | 1.0 | Loss | 17 | p12 | p11.2 | 1.090.366 | 17 |
| MY709.1 | 1.0 | Loss | 17 | p11.2 | p11.2 | 1179.14 | 18 |
| MY709.1 | 3.0 | Gain | 17 | q21.31 | q21.31 | 571.816 | 8 |
| MY709.1 | 3.0 | Gain | 17 | q21.31 | q21.31 | 571.816 | 8 |
| MY709.2 | 1.0 | Loss | 1 | p36.32 | p35.2 | 26.214.448 | 372 |
| MY709.2 | 1.0 | Loss | 1 | p36.32 | p35.2 | 26.214.448 | 372 |
| MY709.2 | 1.0 | Loss | 1 | q21.1 | q21.1 | 599.74 | 21 |
| MY709.2 | 1.0 | Loss | 1 | q21.1 | q21.1 | 599.74 | 21 |
| MY709.2 | 1.0 | Loss | 1 | q21.2 | q21.2 | 845.843 | 8 |
| MY709.2 | 1.0 | Loss | 2 | p25.3 | p11.2 | 89.115.336 | 564 |
| MY709.2 | 1.0 | Loss | 2 | p25.3 | p11.2 | 89.115.336 | 564 |
| MY709.2 | 1.0 | Loss | 3 | q25.1 | q25.33 | 11.336.109 | 81 |
| MY709.2 | 1.0 | Loss | 3 | q25.1 | q25.33 | 11.336.109 | 81 |
| MY709.2 | 1.0 | Loss | 5 | p15.33 | p15.32 | 1.715.291 | 3 |
| MY709.2 | 1.0 | Loss | 5 | p15.33 | p15.32 | 1.715.291 | 3 |
| MY709.2 | 1.0 | Loss | 11 | p11.12 | q12.2 | 10.312.123 | 150 |
| MY709.2 | 1.0 | Loss | 11 | p11.12 | q12.2 | 10.312.123 | 150 |
| MY709.2 | 1.0 | Loss | 11 | q13.1 | q13.1 | 384.338 | 19 |
| MY709.2 | 1.0 | Loss | 11 | q13.1 | q13.1 | 384.338 | 19 |
| MY709.2 | 1.0 | Loss | 11 | q13.4 | q25 | 61.525.457 | 475 |
| MY709.2 | 1.0 | Loss | 11 | q13.4 | q25 | 61.525.457 | 475 |
| MY709.2 | 3.0 | Gain | 14 | q32.33 | q32.33 | 432.785 | 3 |
| MY709.2 | 3.0 | Gain | 14 | q32.33 | q32.33 | 432.785 | 3 |
| MY709.2 | 3.0 | Gain | 16 | p11.2 | p11.1 | 260.15 | 3 |
| MY709.2 | 3.0 | Gain | 16 | p11.2 | p11.1 | 260.15 | 3 |
| MY709.2 | 3.0 | Gain | 17 | q21.31 | q21.31 | 559.309 | 8 |
| MY709.2 | 3.0 | Gain | 17 | q21.31 | q21.31 | 559.309 | 8 |
| MY709.3 | 1.0 | Loss | 1 | q21.1 | q21.1 | 421.711 | 19 |
| MY709.3 | 1.0 | Loss | 1 | q21.1 | q21.1 | 421.711 | 19 |
| MY709.3 | 1.0 | Loss | 1 | q42.2 | q44 | 14.112.725 | 80 |
| MY709.3 | 1.0 | Loss | 1 | q42.2 | q44 | 14.112.725 | 80 |
| MY709.3 | 1.0 | Loss | 2 | q33.2 | q33.2 | 366.916 | 4 |
| MY709.3 | 1.0 | Loss | 2 | q33.2 | q33.2 | 366.916 | 4 |
| MY709.3 | 1.0 | Loss | 4 | q31.3 | q31.3 | 457.018 | 3 |
| MY709.3 | 1.0 | Loss | 4 | q31.3 | q31.3 | 457.018 | 3 |
| MY709.3 | 1.0 | Loss | 11 | q11 | q11 | 307.832 | 0 |
| MY709.3 | 1.0 | Loss | 11 | q11 | q11 | 307.832 | 0 |
| MY709.3 | 1.0 | Loss | 12 | q14.3 | q14.3 | 281.515 | 1 |
| MY709.3 | 1.0 | Loss | 12 | q14.3 | q14.3 | 281.515 | 1 |
| MY709.3 | 1.0 | Loss | 12 | q14.3 | q24.11 | 42.794.049 | 234 |
| MY709.3 | 1.0 | Loss | 12 | q14.3 | q24.11 | 42.794.049 | 234 |
| MY709.3 | 3.0 | Gain | 14 | q32.33 | q32.33 | 530.425 | 3 |
| MY709.3 | 3.0 | Gain | 14 | q32.33 | q32.33 | 530.425 | 3 |
| MY709.3 | 3.0 | Gain | 16 | p11.2 | p11.1 | 284.519 | 3 |
| MY709.3 | 3.0 | Gain | 16 | p11.2 | p11.1 | 284.519 | 3 |
| MY709.4 | 3.0 | Gain | 1 | p21.1 | p21.1 | 203.103 | 6 |
| MY709.4 | 1.0 | Loss | 1 | q21.1 | q21.1 | 550.386 | 21 |
| MY709.4 | 1.0 | Loss | 1 | q21.1 | q21.1 | 550.386 | 21 |
| MY709.4 | 1.0 | Loss | 1 | q21.2 | q21.2 | 650.24 | 7 |
| MY709.4 | 1.0 | Loss | 7 | q11.22 | q34 | 72513.81 | 561 |
| MY709.4 | 1.0 | Loss | 7 | q11.22 | q34 | 72513.81 | 561 |
| MY709.4 | 1.0 | Loss | 7 | q34 | q36.3 | 12.977.534 | 137 |
| MY709.4 | 1.0 | Loss | 7 | q34 | q36.3 | 12.977.534 | 137 |
| MY709.4 | 1.0 | Loss | 7 | q36.3 | q36.3 | 3.643.985 | 22 |
| MY709.4 | 1.0 | Loss | 7 | q36.3 | q36.3 | 3.643.985 | 22 |
| MY709.4 | 1.0 | Loss | 11 | q11 | q11 | 307.718 | 0 |
| MY709.4 | 1.0 | Loss | 12 | q12 | q13.2 | 12.840.028 | 243 |
| MY709.4 | 1.0 | Loss | 12 | q12 | q13.3 | 13.435.813 | 273 |
| MY709.4 | 1.0 | Loss | 12 | q13.2 | q13.3 | 395.541 | 19 |
| MY709.4 | 1.0 | Loss | 12 | q14.3 | q14.3 | 633.256 | 4 |
| MY709.4 | 1.0 | Loss | 12 | q14.3 | q14.3 | 633.256 | 4 |
| MY709.4 | 3.0 | Gain | 14 | q32.33 | q32.33 | 544.951 | 3 |
| MY709.4 | 3.0 | Gain | 14 | q32.33 | q32.33 | 544.951 | 3 |
| MY709.4 | 3.0 | Gain | 16 | p11.2 | p11.1 | 284.519 | 3 |
| MY709.4 | 3.0 | Gain | 16 | p11.2 | p11.1 | 284.519 | 3 |
| MY709.4 | 1.0 | Loss | 18 | p11.32 | p11.31 | 4601.69 | 27 |
| MY709.4 | 1.0 | Loss | 18 | p11.32 | p11.31 | 4601.69 | 27 |
| MY709.4 | 3.0 | Gain | 18 | p11.22 | p11.22 | 517.569 | 3 |
| MY709.4 | 3.0 | Gain | 18 | p11.22 | p11.21 | 1.759.396 | 6 |
| MY709.4 | 3.0 | Gain | 18 | p11.22 | p11.21 | 222.105 | 1 |
| MY709.4 | 3.0 | Gain | 18 | p11.21 | p11.21 | 590.091 | 1 |
| MY709.4 | 3.0 | Gain | 22 | q11.22 | q11.22 | 360.005 | 6 |
